# Incidence and profile of benign epithelial tumors of salivary glands from a single center in Northeast of Brazil

**DOI:** 10.4317/medoral.24056

**Published:** 2020-11-28

**Authors:** Breno Washington Joaquim de Santana, Leorik Pereira da Silva, Marianna Sampaio Serpa, Mariana de Albuquerque Borges, Sérgio Ricardo Soares de Moura, Márcia Maria Fonseca da Silveira, Ana Paula Veras Sobral

**Affiliations:** 1 DDS, Research collaborator, School of Dentistry, University of Pernambuco, Camaragibe, PE, Brazil; 2 DDS, PhD, Adjunct Professor, Institute of Health and Biotechnology, Federal University of Amazonas, Coari, AM, Brazil; 3 DDS, PhD, A.C.Camargo Cancer Center, São Paulo - SP, Brazil; 4 MD, Staff Pathologist, Department of Pathology, Cancer Hospital of Pernambuco, Recife, PE, Brazil; 5 DDS, PhD, Associate Professor, Postgraduate Program in Dentistry, School of Dentistry, University of Pernambuco, Camaragibe, PE, Brazil

## Abstract

**Background:**

Benign tumors of the salivary glands are a group of lesions with varied histopathological and clinical spectrum. The aim was to determine the incidence and clinicopathological characteristics of benign salivary gland neoplasms diagnosed between 2007 and 2016 in a single center located in northeastern Brazil.

**Material and Methods:**

Records regarding sex, age, anatomical location, histopathological subtype and treatment were retrieved, and data were analyzed using the Stata/IC software (version 12.0).

**Results:**

There were above 7,100 cases of neoplasms in the head and neck region, of which 403 corresponded to salivary gland neoplasms. Of these, 238 (59%) were benign, being pleomorphic adenoma (PA) the most frequent neoplasm (n=178; 74.8%), followed by Warthin's tumor (WT) (n=23; 9.7%). Overall, most cases occurred in females (n=136; 57.1%) and age ranged from 11 to 83 years. The parotid gland (n=188; 79%) was the most common anatomical site, and all patients were treated by surgical excision. Of the cases diagnosed as PA, malignant transformation to carcinoma ex-pleomorphic adenoma (CAEXPA) occurred in 7 (3.9%) cases.

**Conclusions:**

The present study confirmed the clinical and demographic profile of benign salivary gland neoplasms, which contributes to the continuous knowledge of current data about these lesions.

** Key words:**Salivary gland, benign neoplasms, epidemiology.

## Introduction

Salivary gland neoplasms represent about 3-6% of all head and neck neoplasms and their annual global incidence is approximately 0.4-13.5 per 100,000 people. Most cases are benign, and present great histopathological diversity. Currently, the World Health Organization (WHO) recognizes 11 subtypes of benign epithelial tumors of the salivary glands, though, some variants such as lymphadenoma, sebaceous adenoma and ductal papilloma are extremely rare (1-7). Large series report that approximately 64-80% of primary neoplasms of epithelial origin occur in the parotid, 7-11% in the submandibular gland, less than 1% in the sublingual gland and 9-23% in minor salivary glands (2-8).

As for their nature, usually more than 50% are benign, reaching a prevalence up to 86% (7). Most cases occur between the fourth and seventh decades of life and women are more affected (2-7). Pleomorphic adenoma (PA) is the most common benign salivary gland neoplasm, comprehending about 50% of the cases, and Warthin's tumor (WT) is the second one, representing 4 to 15% of all salivary gland neoplasms (2-6,8-11). Furthermore, benign neoplasms with enduring evolution and history of recurrences can undergo malignant transformation. For instance, PA can evolve to carcinoma ex-pleomorphic adenoma (CAEXPA), which diminishes the survival of the patient (12).

Epidemiological studies that assess the incidence and characteristics of these tumors help to update scientists and health professionals worldwide. Thus, this retrospective study aimed to analyze the cases of benign salivary gland neoplasms in a referral hospital in northeastern Brazil in order to keep data renewed.

## Material and Methods

The study was evaluated and approved by the local Research Ethics Committee (REC/HCP - no 2.774.008). Medical records of patients attended between 2007 and 2016 at the Cancer Hospital of Pernambuco were retrieved, and for those diagnosed with benign salivary gland neoplasm, clinicopathological characteristics (sex, age, anatomical location, histopathological subtype and treatment performed) were collected. Records had to report histopathological diagnosis of salivary gland neoplasm and all clinical data to be included in the study.

The cases diagnosed as benign salivary gland neoplasms were re-assessed by an oral and maxillofacial pathologist in order to confirm and adequate final histological diagnosis with the current WHO classification (1). For this, 5μm sections of each case were stained with hematoxylin and eosin and evaluated under light microscopy. Nomenclature and criteria used to reclassify the cases were based on the descriptions summarized by the current WHO classification of salivary gland tumors (1). When in doubt about the diagnosis, a second pathologist was consulted. There were no disagreements related to the reclassification and no case was excluded. After histological evaluation, 3 cases previously denominated “oxyphilic adenoma” were renamed as oncocytoma, and 9 cases diagnosed as “monomorphic adenoma” were reclassified to canalicular adenoma (1 case) and the other 8 to basal cell adenoma. In addition, 6 cases of PA were reclassified to myoepithelioma.

Database was mounted and analyzed using the Stata/IC software (version 12.0; StataCorp, CollegeStation, TX), with a descriptive analysis of the sex, age, anatomical location and treatment. The results obtained were assigned to a Table with their respective absolute and relative frequencies.

## Results

In nine years, approximately 230,635 lesions were diagnosed in the referred hospital. Of these, approximately 7,100 were cases of head and neck neoplasms, of which 403 (5.7%) were of salivary gland neoplasms. There were 238 cases of benign salivary gland neoplasm, which corresponded to 59% of all salivary gland neoplasms and 3.3% of the head and neck tumors. Most cases were diagnosed in females (n=136; 57.1%) and age varied between 11 and 83 years, with an average of 48 years-old (SD±16). PA was the most frequent neoplasm (n=178; 74.8%), followed by WT (n=23; 9.7%) (Table 1).

**Table 1:**
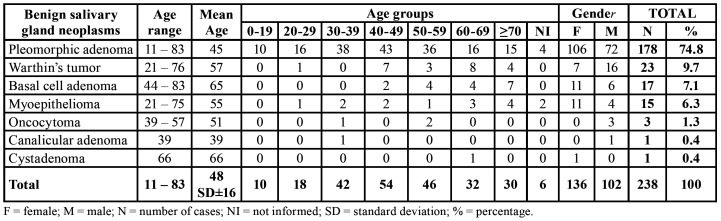
Age group and gender distribution of benign salivary gland neoplasms

**Figure 1:**
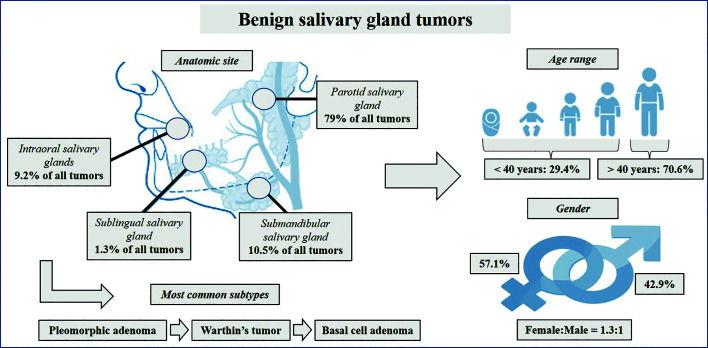
General clinicopathological findings of benign salivary gland neoplasms.

Benign salivary gland neoplasms occurred more in major salivary glands (n=216; 90.8%), with the parotid gland being the most common anatomical site (n=188; 79%) and PA the most predominant tumor. WT (n=23; 9.7%), oncocytoma (n=3; 1.3%) and cystadenoma (n=1; 0.4%) affected only the parotid gland. Furthermore, most cases of basal cell adenoma (n=16; 6.7%) and myoepithelioma (n=12; 5%) also occurred in this anatomical site. Minor salivary glands represented 9.2% (n=22) of the cases, with the palate (n=13; 5.5%) being the most frequent region, followed by the buccal mucosa (n=6; 2.5%). The most common histological diagnosis at both sites was PA (Table 2). Fig. 1 illustrates the main findings of this study.

Patients with lesions in the major salivary glands were submitted to fine-needle aspiration prior to surgical treatment, in order to obtain the diagnosis. Seven cases diagnosed as PA and 2 cases diagnosed as WT were considered malignant after surgical resection and histopathological analysis. Mucoepidermoid carcinoma, adenoid cystic carcinoma and unspecified adenocarcinoma were the definitive diagnoses. As for the tumors located in the minor salivary glands, incisional biopsy was conducted for diagnosis prior to surgical excision.

All patients diagnosed with benign salivary gland neoplasms performed a non-radical surgical excision. Preservation of the facial nerve was maintained for all surgeries of the parotid gland. Of the 178 cases diagnosed as PA, malignant transformation to CAEXPA occurred in 7 cases (3.9%). These cases had a history of PA with one or two recurrences before the transformation and no case was initially diagnosed as malignant (primary CAEXPA). Evolution time until the diagnosis of CAEXPA ranged from 2 to 10 years. Table 3 shows that for previous studies conducted in Brazil the rate of malignant transformation of PA varied from 0-10.8% in several diagnostic centers.

**Table 2:**
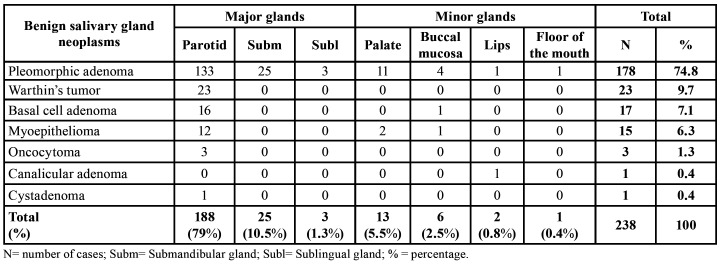
Anatomical site distribution of benign salivary gland neoplasms.

**Table 3:**
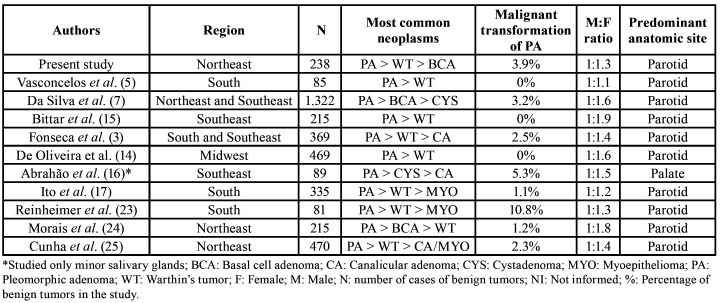
General features of benign salivary gland neoplasms from studies conducted in Brazil (2005-2020).

## Discussion

In the studied hospital, benign salivary gland neoplasms (n=238; 59%) represented the majority of the cases, being in agreement with worldwide literature (2-15). Furthermore, other studies conducted in Brazil also confirm our findings (3,5,7,14,16,17). In the present study, the average age was 48 years-old (SD ± 16), with a range from 11 to 83 years. Most cases occurred in females and the parotid gland was the most frequent site representing 79% of the cases. Gao *et al*. (6) and Da Silva *et al*. (7) found similar results to ours were most cases of benign salivary gland neoplasm occurred in the 5th decade of life, females and parotid gland. In contrast, two studies conducted by Tian *et al*. (2) and Wang *et al*. (4) found that the male gender was most affected by salivary tumors. This indicates that some divergence may be found in the literature, reinforcing the importance to perform epidemiological studies worldwide and compare their results.

PA was the most common benign neoplasm, representing about 75% of cases, followed by WT with 9.7%. Corroborating these findings, other studies showed similar results (2-6,8-11,14-16). Nevertheless, in a multicenter study conducted in Brazil, Da Silva *et al*. (7) observed basal cell adenoma was the second most prevalent tumor followed by cystadenomas and myoepitheliomas. According to this author, WT may be even more scarce than indicated by the literature. Furthermore, apart from this referred study, other two conducted in Brazil, showed WT is not always the second most frequent tumor showing some discrepancy even in the same country (Table 3).

In our analysis, WT affected exclusively the parotid gland with the majority of the cases occurring in males. According to the studies of Ito *et al*. (17), Oliveira *et al*. (14), Tian *et al*. (2), Bello *et al*. (13), Fonseca *et al*. (3), Wang *et al*. (4), Vasconcelos *et al*. (5), Gao *et al*. (6), Pinheiro *et al*. (18) and Shen *et al*. (9), the parotid gland represents the most prevalent anatomical site of WT with men being the most affected. Tian *et al*. (2), highlighted an increase in the incidence of WT in females during the last 50 years and a change in the male:female ratio from 10:1 in 1953 to 1.2:1 in 1996. This may be associated with the increase in the number of women with smoking habits in the last decades, which is a risk factor for this lesion.

Only three cases of sublingual glands were observed, in which all were diagnosed as PA. This anatomical site is the most unusual location for benign salivary gland neoplasms. In fact, Ito *et al*. (17), Oliveira *et al*. (14), Fonseca *et al*. (3) and González *et al*. (8) did not report any involvement of the sublingual gland by benign neoplasms in their studies. Associated with this, we believe that there is a strong tendency that, when affected, the sublingual gland is mainly a site of malignant tumors.

Benign neoplasms in minor glands were more prevalent on the palate, followed by the buccal mucosa. PA was the most frequent lesion at both sites, being reported similar results by Wang *et al*. (4) and Gao *et al*. (6). Studies that exclusively addressed tumors in minor salivary glands are also in agreement with our results regarding the most common anatomical site and histological diagnosis. However, they showed variations regarding the second most common anatomical site in which they reported being the lips or tongue instead of the buccal mucosa (16,19,20).

In our sample, all cases were treated by non-radical surgical excision. Vasconcelos *et al*. (5), Dalgic *et al*. (20), Rapidis *et al*. (21) and Sun *et al*. (22), also reported that surgery was the treatment of choice for benign salivary gland neoplasms. Overall, there are limited studies published in the literature that present data concerning the treatment of these tumors. This is attributed to the fact that surgery is the gold standard therapy for benign salivary gland neoplasms, being this treatment already very well-established.

CAEXPA is a neoplasm with an epithelial and/or myoepithelial component that develops from primary PA or recurrent tumors. It represents approximately 3.6% of all salivary gland neoplasms and 12% of the malignant salivary gland neoplasms (1,12). Larger series studies have revealed variations in the occurrence of CAEXPA. In a research conducted by Tian *et al*. (2), there were 3,281 cases of PA and 179 cases of CAEXPA, which constituted 5.4% of PA malignant transformation. In general, other authors indicate that nearly 2.5 to 6.8% of all PA present malignant transformation (4,6,10). As showed in Table 3, studies in Brazil show variation in the percentage of CAEXPA, however, the transformation rate in general is between 1 and 5% (3,7,17,23-25). In our analysis, of the 178 cases diagnosed as PA, there was malignant transformation in 7 patients, representing 3.9% of the cases. History of recurrences of PA prior to the transformation was noted confirming an important characteristic associated with the development of CAEXPA (12).

An important aspect to consider is that the tumors analyzed derived from a medical pathology center (general pathology service), instead of an oral pathology service. It has been shown (3,25) that in medical services the most common location for benign salivary gland neoplasms is the parotid gland in which PA and WT are the most frequent tumors as we observed in the present study. In the other hand, in an oral pathology laboratory most lesions diagnosed are from the minor salivary glands, especially the palate, evidencing a different distribution profile. Furthermore, PA is also the most common tumor in this location, followed by other tumors such as canalicular adenoma, myoepithelioma and basal cell adenoma. The fact that this study was conducted in a medical center also indicates that WT was the second most common tumor as it occurs almost exclusively in the parotid gland as already discussed. Thereby, when analyzing the distribution of salivary gland tumors, the pathology service (general or oral) the lesions were evaluated has to be taken into consideration as it influences the clinicopathological profile of these tumors.

## Conclusions

Our results were similar to those found in the literature. PA was the most common benign neoplasm, occurring mainly in the parotid gland and female patients. Considering the histopathological variety of salivary gland neoplasms, this study contributed to update current knowledge about the clinicopathological characteristics of these tumors, emphasizing its continuous importance for scientific and clinical purposes.

## References

[1] Seethala RR, Stenman G (2017). Update from the 4th Edition of the World Health Organization Classification of Head and Neck Tumours: Tumors of the Salivary Gland. Head Neck Pathol.

[2] Tian Z, Li L, Wang L, Hu Y, Li J (2010). Salivary gland neoplasms in oral and maxillofacial regions: a 23-year retrospective study of 6982 cases in an eastern Chinese population. Int J Oral Maxillofac Surg.

[3] Fonseca FP, Carvalho Mde V, de Almeida OP, Rangel AL, Takizawa MC, Bueno AG (2012). Clinicopathologic analysis of 493 cases of salivary gland tumors in a Southern Brazilian population. Oral Surg Oral Med Oral Pathol Oral Radiol.

[4] Wang XD, Meng LJ, Hou TT, Huang SH (2015). Tumours of the salivary glands in northeastern China: a retrospective study of 2508 patients. Br J Oral Maxillofac Surg.

[5] Vasconcelos AC, Nör F, Meurer L, Salvadori G, Souza LB, Vargas PA (2016). Clinicopathological analysis of salivary gland tumors over a 15-year period. Braz Oral Res.

[6] Gao M, Hao Y, Huang MX, Ma DQ, Chen Y, Luo HY (2017). Salivary gland tumours in a northern Chinese population: a 50-year retrospective study of 7190 cases. Int J Oral Maxillofac Surg.

[7] da Silva LP, Serpa MS, Viveiros SK, Sena DAC, de Carvalho Pinho RF, de Abreu Guimarães LD (2018). Salivary gland tumors in a Brazilian population: A 20-year retrospective and multicentric study of 2292 cases. J Craniomaxillofac Surg.

[8] Campolo González A, Ramírez Skinner H, Vargas Díaz A, León Ramírez A, Goñi Espildora I, Solar González A (2018). Epithelial tumors of salivary glands. Review of 286 pathology reports. Rev Med Chil.

[9] Shen SY, Wang WH, Liang R, Pan GQ, Qian YM (2018). Clinicopathologic analysis of 2736 salivary gland cases over a 11-year period in Southwest China. Acta Otolaryngol.

[10] Sentani K, Ogawa I, Ozasa K, Sadakane A, Utada M, Tsuya T (2019). Characteristics of 5015 Salivary Gland Neoplasms Registered in the Hiroshima Tumor Tissue Registry over a Period of 39 Years. J Clin Med.

[11] Mejía-Velázquez CP, Durán-Padilla MA, Gómez-Apo E, Quezada-Rivera D, Gaitán-Cepeda LA (2012). Tumors of the salivary gland in Mexicans. A retrospective study of 360 cases. Med Oral Patol Oral Cir Bucal.

[12] Mariano FV, Noronha AL, Gondak RO, Altemani AM, de Almeida OP, Kowalski LP (2013). Carcinoma ex pleomorphic adenoma in a Brazilian population: clinico-pathological analysis of 38 cases. Int J Oral Maxillofac Surg.

[13] Bello IO, Salo T, Dayan D, Tervahauta E, Almangoush A, Schnaiderman-Shapiro A (2012). Epithelial salivary gland tumors in two distant geographical locations, Finland (Helsinki and Oulu) and Israel (Tel Aviv): a 10-year retrospective comparative study of 2,218 cases. Head Neck Pathol.

[14] de Oliveira FA, Duarte EC, Taveira CT, Máximo AA, de Aquino EC, Alencar Rde C (2009). Salivary gland tumor: a review of 599 cases in a Brazilian population. Head Neck Pathol.

[15] Bittar RF, Ferraro HP, Moraes Gonçalves FT, Couto da Cunha MG, Biamino ER (2015). Neoplasms of the salivary glands: analysis of 727 histopathological reports in a single institution. Otolaryngol Pol.

[16] Abrahão AC, Santos Netto Jde N, Pires FR, Santos TC, Cabral MG (2016). Clinicopathological characteristics of tumours of the intraoral minor salivary glands in 170 Brazilian patients. Br J Oral Maxillofac Surg.

[17] Ito FA, Ito K, Vargas PA, de Almeida OP, Lopes MA (2005). Salivary gland tumors in a Brazilian population: a retrospective study of 496 cases. Int J Oral Maxillofac Surg.

[18] Pinheiro J, Sá Fernandes M, Pereira AR, Lopes JM (2018). Histological Subtypes and Clinical Behavior Evaluation of Salivary Gland Tumors. Acta Med Port.

[19] Dhanuthai K, Boonadulyarat M, Jaengjongdee T, Jiruedee K (2009). A clinico-pathologic study of 311 intra-oral salivary gland tumors in Thais. J Oral Pathol Med.

[20] Dalgic A, Karakoc O, Aydin U, Hidir Y, Gamsizkan M, Karahatay S (2014). Minor salivary gland neoplasms. J Craniofac Surg.

[21] Rapidis AD, Stavrianos S, Lagogiannis G, Faratzis G (2004). Tumors of the submandibular gland: clinicopathologic analysis of 23 patients. J Oral Maxillofac Surg.

[22] Sun G, Yang X, Tang E, Wen J, Lu M, Hu Q (2010). The treatment of sublingual gland tumours. Int J Oral Maxillofac Surg.

[23] Reinheimer A, Vieira DS, Cordeiro MM, Rivero ER (2019). Retrospective study of 124 cases of salivary gland tumors and literature review. J Clin Exp Dent.

[24] Morais Mde L, Azevedo PR, Carvalho CH, Medeiros L, Lajus T, Costa Ade L (2011). Clinicopathological study of salivary gland tumors: an assessment of 303 patients. Cad Saude Publica.

[25] Cunha JL, Coimbra AC, Silva JV, Nascimento IS, Andrade ME, Oliveira CR (2020). Epidemiologic analysis of salivary gland tumors over a 10-years period diagnosed in a northeast Brazilian population. Med Oral Patol Oral Cir Bucal.

